# Probiotics and Their Antimicrobial Metabolites: A Collegial Strategy for Food Bio‐Preservation – A Review

**DOI:** 10.1002/fsn3.71318

**Published:** 2025-12-04

**Authors:** Lingling Wang, Shuanshan Ren, Atique Ahmed Behan, Muhammad Asif Arain, Nissar Ahmed Ujjan, Dequan Zeng, Yufeng Li, Xingming Ma

**Affiliations:** ^1^ School of Comprehensive Health Management Xihua University Chengdu China; ^2^ Department of Animal and Veterinary Sciences, College of Agricultural and Marine Sciences Sultan Qaboos University Muscat Oman; ^3^ Faculty of Veterinary and Animal Sciences Lasbela University of Agriculture, Water and Marine Sciences Balochistan Pakistan

**Keywords:** antimicrobial proteins, bio‐preservation, food safety, probiotics, shelf life extension

## Abstract

Ensuring food safety and extending the shelf life of perishable products remain major challenges for the global food industry. The escalating consumer demand for natural food preservatives has intensified research into biological alternatives to chemical additives. Probiotics, traditionally recognized for their health‐promoting effects, have recently gained attention for their capacity to improve food preservation. Beyond their direct competitive exclusion of pathogens, probiotics produce a wide range of antimicrobial peptides (AMPs), including bacteriocins, enzymes, and peptide‐based inhibitors, which exhibit potent activity against foodborne spoilage and pathogenic microorganisms. Individually, both probiotics and their AMPs have been explored as natural biopreservatives; however, growing evidence suggests that their synergistic application offers enhanced efficacy. The synergy arises from the multi‐targeted approach: probiotics colonize the food matrices, creating a protective biofilm, while simultaneously producing a spectrum of AMPs that disrupt bacterial cell membranes, inhibit cell wall synthesis, and suppress virulence gene expression. This combination extends the shelf life of various food products, including dairy, meats, and vegetables, by effectively controlling microbial load and delaying spoilage. Furthermore, the use of probiotic‐derived AMPs, often exhibiting thermal and pH stability, offers a natural label‐friendly option for the food industry. Despite these advancements, challenges remain regarding strain specificity, scalability, regulatory approval, and sensory impacts on food products. This review comprehensively examines the mechanisms of action, efficacy in various food matrices, and potential for industrial application, concluding that the strategic integration of probiotics and their antimicrobial proteins represents a potent, natural, and synergistic strategy for ensuring food safety and sustainability.

## Graphical abstract

Illustrates the potential of probiotics and their antimicrobial metabolites in food bio‐preservation.

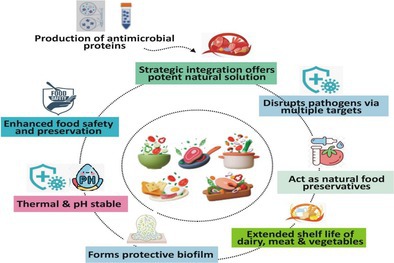

## 1 Introduction

The integrity of the global food chain is fundamentally dependent on effective safety and preservation strategies. Foodborne illnesses, caused by pathogens such as *Salmonella* spp., *Listeria monocytogenes* , and *Escherichia coli* , remain a severe public health burden, causing an estimated 600 million cases and 420,000 deaths annually worldwide (Ndondo 2023). Furthermore, post‐harvest spoilage due to microbial activity leads to massive economic losses and contributes significantly to global food waste, a critical concern for food security and environmental sustainability (Arain, Khaskheli, et al. 2023; Gustavsson et al. 2011). Therefore, developing robust methods to inhibit the growth of pathogenic and spoilage microorganisms is paramount for protecting consumer health and ensuring the stability and longevity of the food supply.

Conventional preservation methods, including thermal processes, addition of chemical preservatives, dehydration and refrigeration, have been widely used to extend shelf life and ensure food safety. However, these approaches often present considerable limitations, as thermal treatments can compromise the sensory, nutritional, and functional properties of food products, while chemical additives may raise health safety concerns among consumers (Arain, Salman, et al. 2024; Arain, Khaskheli, et al. 2025). The application of chemical preservatives (nitrites, sulfites, and organic acids) has raised consumer concerns regarding potential health risks, including allergic reactions and the association of some compounds with chronic diseases (Arain, Rasheed, et al. 2023; Silva and Lidon 2016). More critically, the extensive and often indiscriminate use of antibiotics in agriculture and chemical preservatives has accelerated the emergence of multidrug‐resistant (MDR) pathogens, posing a formidable threat to global health (Saeed et al. 2021; Nabi et al. 2020). This growing resistance, coupled with increasing consumer demand for “clean‐label,” natural, and minimally processed foods, has necessitated the exploration of novel, sustainable, and safe alternatives from natural sources (Ashraf et al. 2024; Arain, Nabi, Shah, et al. 2022).

In this context, biocontrol using beneficial microorganisms and their natural metabolites has emerged as a highly promising strategy. Probiotics, defined as “live microorganisms that, when administered in adequate amounts, confer a health benefit on the host,” have been extensively studied for their gut health benefits (Staniszewski and Kordowska‐Wiater 2021; Hill et al. 2014). However, their role extends beyond the host; many probiotic strains, particularly lactic acid bacteria (LAB) and *Bifidobacteria*, are potent producers of a diverse arsenal of antimicrobial peptides (AMPs), most notably bacteriocins (Tagliazucchi et al. 2019; Mokoena 2017). These ribosomally synthesized peptides or proteins exhibit potent and often broad‐spectrum bacteriostatic activity against closely related bacterial strains, including many foodborne pathogens and spoilage organisms, with minimal impact on the surrounding microbiota or host cells (Saeed et al. 2024; Soltani et al. 2021). The synergistic interaction between specific probiotic strains and their associated antimicrobial metabolites such as bacteriocins, provides a dual mechanism for enhancing food safety. Probiotics exert beneficial effects through competitive exclusion of pathogens, modulation of microbial balance, and production of antimicrobial metabolites. Meanwhile, bacteriocins, when derived from generally recognized as safe (GRAS) microorganisms such as *Lactobacillus*, *Pediococcus*, and *Enterococcus* species offer targeted and biodegradable antimicrobial activity. However, it is important to note that not all bacteriocins are classified as GRAS, and their safety depends on the producing strain and intended application (Choi et al. 2024). Therefore, combinations involving GRAS‐certified probiotics and their corresponding safe bacteriocins (nisin from *Lactococcus lactis* or pediocin from *Pediococcus acidilactici* ) are particularly promising for ensuring regulatory compliance and consumer safety while improving food preservation (Parada Fabián et al. 2025). Although the antimicrobial roles of probiotic cells and purified bacteriocins have been widely reported, their synergistic interplay remains insufficiently explored. Most reviews treat these components separately, emphasizing either competitive exclusion by live probiotics or the direct inhibition mediated by isolated AMPs. This review addresses that gap by critically examining the combined, synergistic mechanisms through which viable probiotic cells and their AMPs contribute to food preservation. We further highlight emerging applications of these bio‐protective systems in dairy and meat products, and discuss key translational barriers including microbial viability, peptide stability, and regulatory constraints that influence their industrial implementation.

## 2 Probiotics in Food Systems: Definition, Strains, and Bio‐Preservative Role

The integration of probiotics into food matrices represents a significant advancement in the field of functional foods, aimed at enhancing human health while simultaneously contributing to the product's shelf‐life and safety. This section delineates the fundamental criteria for probiotics, enumerates the predominant microbial strains employed industrially, and elucidates their functional roles in food preservation.

## 3 Definition of Probiotic

The “probiotic” are living microbes or bioactive microbial entities that, when delivered in viable or functionally active forms and in scientifically validated quantities, beneficially modulate host physiology, metabolism, and microbial ecology to promote health and prevent disease (Rajput et al. 2017; Sun et al. 2017; Morelli 2013). This definition is underpinned by a set of essential criteria that a microbial strain must fulfill to be classified as a probiotic. Firstly, it must be precisely identified at the genus, species, and strain levels using genomic techniques (16S rRNA sequencing) (Hill et al. 2014). Secondly, it must be safe for its intended use, devoid of virulence factors and antibiotic resistance genes that can be horizontally transferred. Thirdly, it must demonstrate resilience to survive passage through the gastrointestinal tract, including tolerance to gastric acidity and bile salts. Finally, and most crucially, it must be supported by robust human clinical trials demonstrating a validated health benefit at a defined dosage, typically requiring a viable count of 10^6^ to 10^7^ colony‐forming units (CFU) per gram or milliliter of the food product at the time of consumption (Binda et al. 2020).

## 4 Probiotic Strains

The most prevalent probiotic strains belong to *Lactobacillus* and *Bifidobacterium*, both dominant commensals of the human gut. Within *Lactobacillus*, species such as *L. acidophilus* , *L. casei* , *L. plantarum* and the clinically validated strain *L. rhamnosus* (LGG), are extensively incorporated into fermented dairy products including yogurt, kefir, and cheese due to their robust survival and well‐documented functional traits (Sánchez et al. 2017). For *Bifidobacterium* strains such as *B. animalis* subsp. *lactis* BB‐12 and *B. longum* , they are frequently used in dairy matrices owing to their documented safety and gut modulatory properties, despite their more fastidious growth and oxygen sensitive (O'callaghan and Van Sinderen 2016). LGG and BB‐12 are cited at the strain level because they represent the most extensively characterized and commercially standardized probiotic strains, each supported by a large body of clinical evidence and regulatory approval in contrast to other *lactobacilli* and *bifidobacteria* that are often referenced only at the species level in the literature.

## 5 Bio‐Preservative Functions

Beyond their acclaimed health‐promoting potential, probiotics serve as natural bio‐preservatives that fortify food systems against spoilage and contamination. Their diverse metabolic activities, encompassing the production of organic acids, bacteriocins, hydrogen peroxide, and other antimicrobial metabolites, directly and indirectly enhance food safety, inhibit pathogenic microorganisms, and extend shelf life. The fermentation process led by lactic acid bacteria (LAB) rapidly acidifies the food environment through the production of lactic acid, lowering the pH to levels that inhibit the growth of many spoilage organisms and foodborne pathogens, such as *Listeria monocytogenes* , *Staphylococcus aureus* , and *Escherichia coli* (Maia et al. 2023). Furthermore, many probiotic strains synthesize ribosomally synthesized antimicrobial peptides called bacteriocins (nisin from *Lactococcus lactis* , plantaricin from *L. plantarum* ), which have a targeted bactericidal action against closely related Gram‐positive bacteria (Silva, Silva, and Ribeiro 2018). This natural preservation system reduces the reliance on chemical additives, aligning with the consumer demand for “clean‐label” products. The competitive exclusion principle also applies, whereby the high density of probiotic bacteria outcompetes potential pathogens for nutrients and adhesion sites, thereby stabilizing the microbial ecology of the food product (Leyva Salas et al. 2017).

## 6 Antimicrobial Proteins from Probiotics; Classification and Mode of Action

Probiotic microorganisms secrete a broad arsenal of antimicrobial biomolecules that collectively mediate pathogen inhibition and microbiome stabilization. These can be broadly categorized into (i) AMPs, an small ribosomally encoded peptides with direct microbicidal activity, and (ii) antimicrobial enzymes, such as lysozyme, proteases, and phospholipases, which exert killing by degrading structural components of pathogens rather than acting via peptide membrane interaction (Wieërs et al. 2020). Within the AMP category, bacteriocins represent a well‐defined subclass: they are ribosomally synthesized AMPs produced by bacteria, often with narrow‐spectrum antagonism against phylogenetically related strains. Thus, bacteriocins fall within the broader AMP group. Their inclusion under AMPs is justified mechanistically and biosynthetically: like canonical AMPs, bacteriocins destabilize microbial membranes or interfere with cellular targets, but they differ in their post‐translational modifications, ecological niche functions, and classification schemes defined for lactic acid bacteria. Therefore, bacteriocins do not constitute a third independent category but rather a taxonomically and functionally distinct subset of AMPs that co‐exist with non‐ribosomal peptide AMPs and enzyme‐mediated antimicrobial systems in probiotic biology.

**FIGURE 1:**
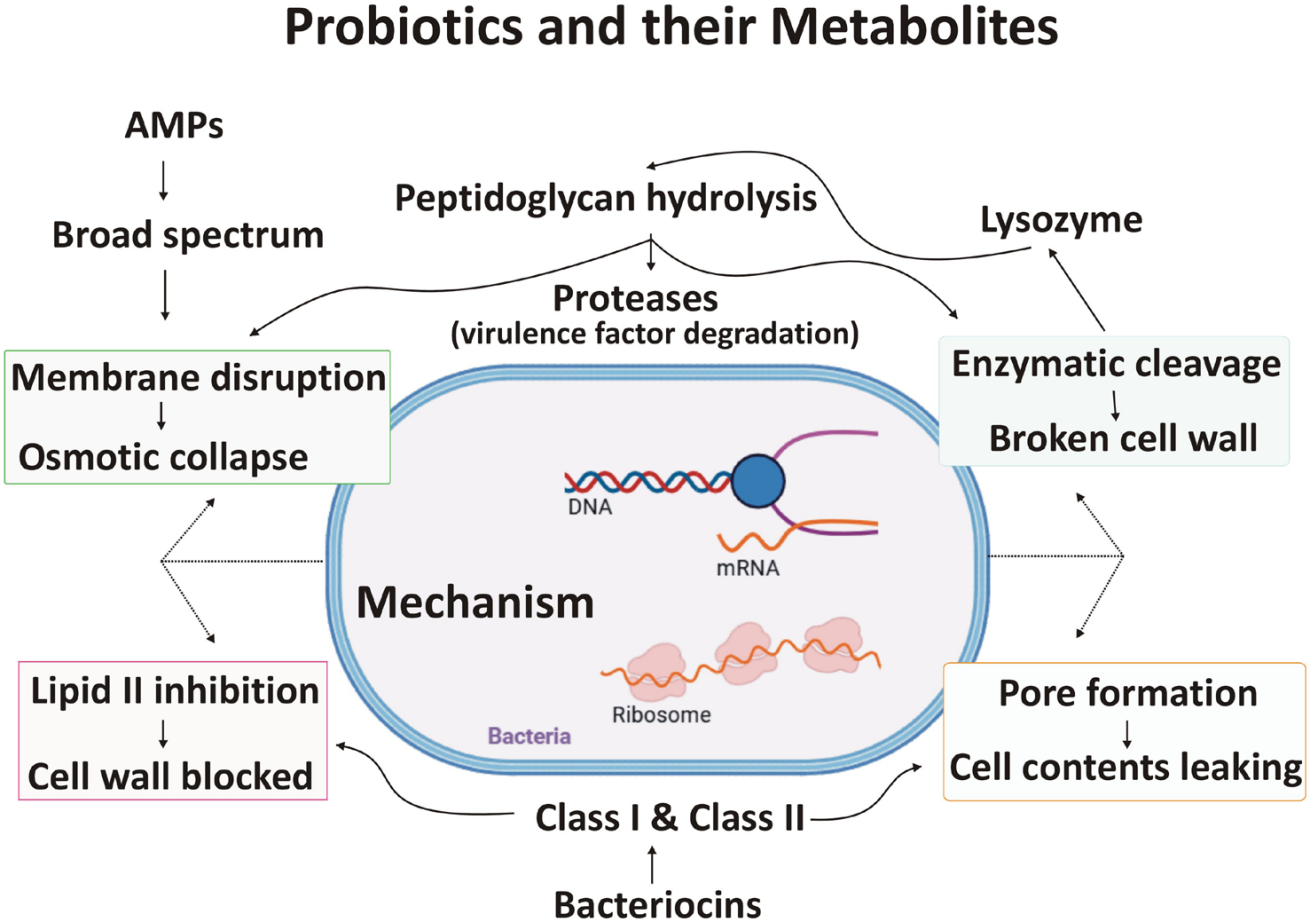
Showing the classification of antimicrobial metabolites produced by probiotics and their mechanisms of bacterial inhibition.

Bacteriocins produced by probiotics are broadly classified into four major classes: Class I (lantibiotics), Class II (non‐lantibiotics), Class III (large heat‐labile proteins), and Class IV (complex bacteriocins associated with lipids or carbohydrates) (Cotter et al. 2013; Mokoena 2017). Class II bacteriocins are additionally subdivided into Class IIa (pediocin‐like peptides with strong anti‐Listeria activity) and Class IIb (two‐peptide bacteriocins requiring both peptides for full activity) (Fimland et al. 2005). Class I bacteriocins, such as nisin from *Lactococcus lactis* , are post‐translationally modified peptides that target lipid II, disrupt cell wall biosynthesis, and form pores in bacterial membranes (Bahrami et al. 2024). By contrast, Class II bacteriocins (e.g., pediocin PA‐1 from *Pediococcus acidilactici* ), which lack extensive post‐translational modifications, mainly kill through membrane permeabilization and generally display a narrow to moderate antimicrobial spectrum against Gram‐positive pathogens (Cintas et al. 1998). Figure 1 schematically depicts the classification framework and canonical modes of action of probiotic‐derived antimicrobial peptides.

Enzymes with antimicrobial properties form a crucial third category. The spectrum of activity is often wider than that of typical bacteriocins, though activity against Gram‐negatives can be enhanced in combination with outer membrane permeabilizers. Lysozyme, produced by certain probiotic strains like some *Bacillus* spp., hydrolyzes the β‐(1,4)‐glycosidic bonds between N‐acetylmuramic acid and N‐acetylglucosamine in peptidoglycan, directly lysing the cell walls of susceptible bacteria (Ibrahim et al. 2001). Proteases and other hydrolytic enzymes can degrade bacterial surface proteins or virulence factors, such as toxins, thereby neutralizing the threat posed by pathogens without direct bactericidal activity (Lebrun et al. 2009). However, the antimicrobial action is highly specific to the enzyme's substrate; lysozyme, for example, is most effective against Gram‐positive bacteria. The synergistic action of these proteins enhances the overall antimicrobial efficacy of probiotics, making them a critical component in maintaining gut homeostasis and preventing infectious diseases.

## 7 Synergistic Interaction Between Probiotics and Their Antimicrobial Metabolites

**FIGURE 2:**
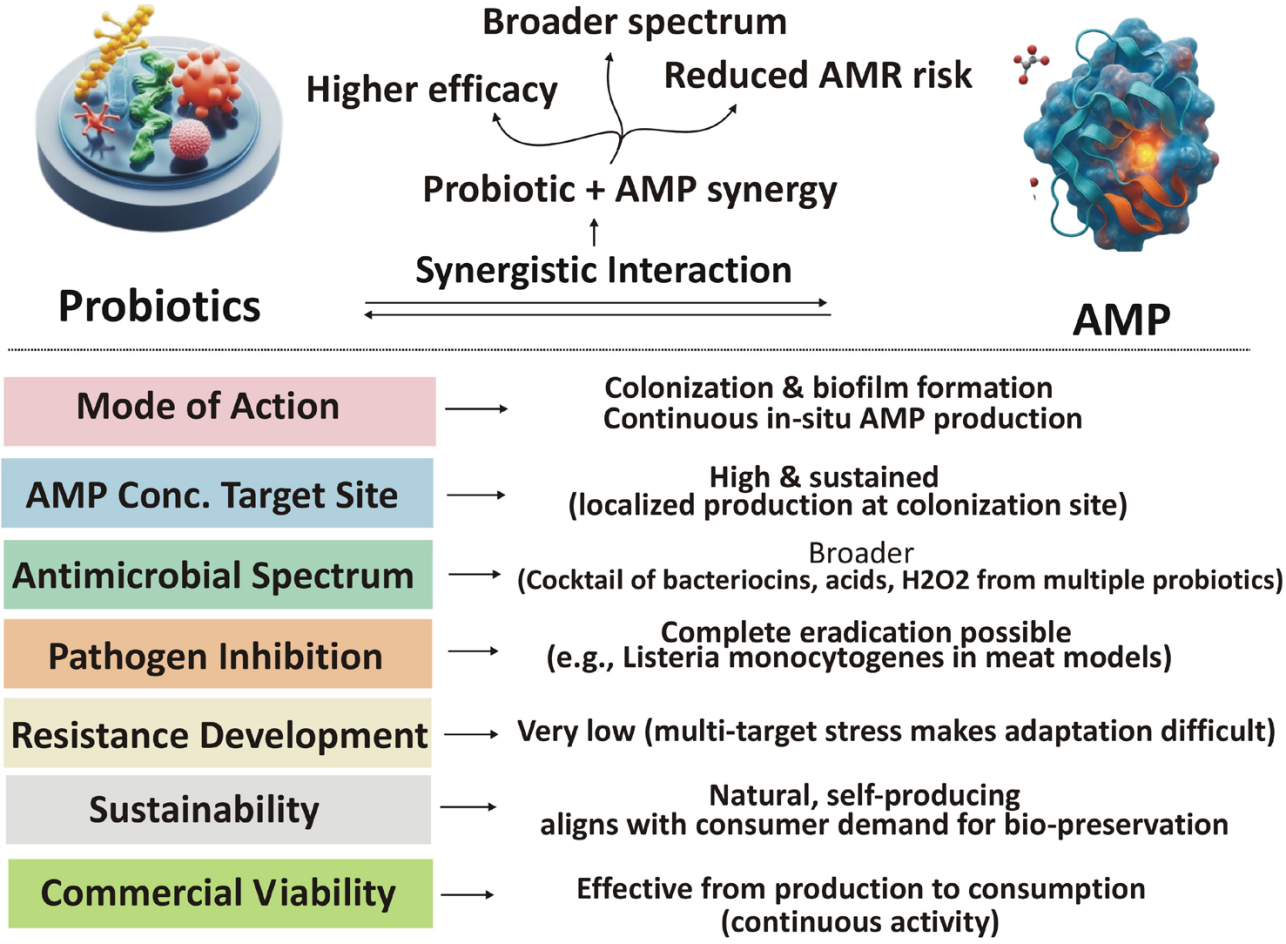
Showing the synergistic interaction between probiotics and antimicrobial proteins for enhanced antimicrobial efficacy and food bio‐preservation.

The antibacterial efficacy of probiotics in food systems does not rely solely on competitive exclusion but is significantly enhanced by their synergistic interaction with secreted metabolites, such as bacteriocins, organic acids, and hydrogen peroxide (Liu et al. 2020). This synergy is mechanistically grounded in a two‐step process: (i) special colonization and biofilm establishment on food matrices or host epithelium, creating a persistent ecological barrier, and (ii) in situ continuous AMPs secretion, ensuring localized high antimicrobial pressure precisely at the pathogen entry zone and effect consistently shown to overtake exogenously applied purified peptides (Gálvez et al. 2007; Mokoena 2017; Zinöcker and Lindseth 2018). Figure 2 showed a detailed overview of synergetic representation of probiotics and AMPs.

The co‐culture studies demonstrate that combining distinct bacteriocinogenic strains not only increases bactericidal magnitude but broadens the inhibitory spectrum. For example, the pairing of *Lactobacillus sakei* CRL1862 (sakacin producer) and *Pediococcus acidilactici* CRL1873 (pediocin producer) 10^6^ CFU/g, yielded complete eradication of *Listeria monocytogenes* in meat systems, whereas each strain alone was only inhibitory (Castellano et al. 2017; Giaouris 2020). This synergistic effect was not solely attributed to bacteriocin activity but also due to the concurrent production of other antimicrobial metabolites, including organic acids (mainly lactic and acetic acids) and hydrogen peroxide, which collectively enhanced the antimicrobial efficacy. Likewise, *Lactobacillus plantarum* AMA‐K exhibited superior anti‐listerial activity in cheese when colonizing as viable cells compared to equivalent dose of cell‐free bacteriocin supernatants, underscoring the role of biofilm‐anchored, self‐renewing delivery (Iranmanesh and Mojgani 2025; Todorov 2008).

This synergy confers multiple strategic advantages. First multi‐targeted convergence (acidification, peroxide stress, pore‐forming peptides, nutrient competition) makes simultaneous microbial adaptation highly improbable (Cotter et al. 2013; Simons et al. 2020). Secondly, the combinational stresses significantly reduce antimicrobial resistance (AMR) emergence, unlike reliance on a single purified bacteriocin (Gálvez et al. 2007; Simons et al. 2020). Finally, such self‐producing “living biopreservative systems” align with clean‐label and sustainability imperatives, enabling pathogen control from processing through consumption without synthetic preservatives.

## 8 Applications in Food Safety and Preservation

Probiotics and their AMPs, have emerged as compelling bio‐preservation tools capable of complementing conventional hurdles such as chemical preservatives, nitrites and thermal sterilization. Compared with synthetic additives probiotic‐derived antimicrobial metabolites provide pathogen control with a lower toxicological burden and preserve sensory and nutritional quality, aligning with clean‐label and minimally processed food trends (Dewi and Kollanoor Johny 2022). The inhibitory action of probiotics is mediated by an integrated metabolite portfolio of low‐molecular‐weight organic acids lowering pH and anionizing membranes, hydrogen peroxide inducing oxidative lethality, competition for nutrients and adhesion sites, and ribosomally synthesized AMPs such as nisin, plantaricin, pediocin, and enterocins that form pores or block cell wall biosynthesis in target pathogens (Davares et al. 2022; Prabhurajeshwar and Chandrakanth 2017).

Bacteriocin‐based preservation provides a mechanistically selective approach that enables targeted inhibition of food‐borne pathogens such as *Listeria monocytogenes* , *Clostridium botulinum* , and *Staphylococcus aureus* without disrupting the desirable fermentative microbiota (Renye Jr and Somkuti 2015). Nisin the most mature example with GRAS/QPS approval is already implemented in cheese and ready‐to‐eat meat matrices as a “front‐runner” success case (Langa et al. 2021). Nonetheless, its antimicrobial performance is constrained by pH, fat content, and proteolytic degradation, necessitating formulation strategies such as encapsulation or rational co‐hurdling with mild heat, modified‐atmosphere packaging, or phytophenolic adjuncts (Bodie et al. 2024). In contrast, multi‐component probiotic fermentates (cell‐free supernatants) deliver a broader inhibitory spectrum through combined organic acids, bacteriocins, and peroxide; however, they suffer from pronounced batch‐to‐batch variability and currently lack clear regulatory pathways for direct food‐additive applications.

**FIGURE 3:**
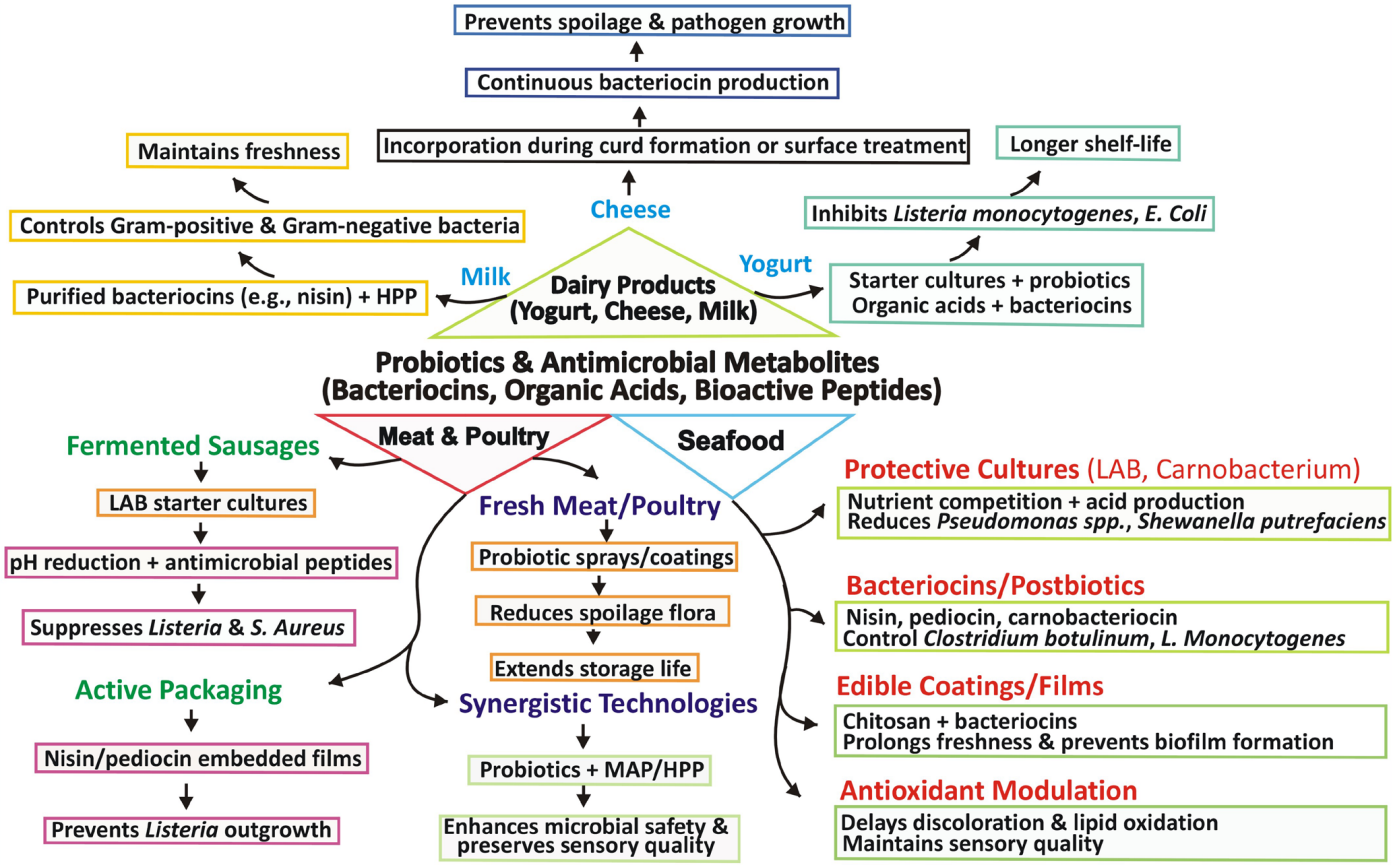
Illustrating the application of probiotics and their antimicrobial proteins in food safety and preservation.

Comparatively, live probiotic inoculation in dairy, vegetables, beverages, and fermented meats contributes both in situ antimicrobial pressure and value‐added health claims; yet, its efficacy depends on strain colonization kinetics, substrate ecology, and oxygen/pH resilience constraints not shared by purified AMPs, which are dosage‐controlled but lack self‐renewal and may require formulation protection. Thus, live cultures and purified AMPs occupy complementary niches: probiotics are superior when concurrent fermentation and health functionality are desired, whereas isolated AMPs are preferable in non‐fermented matrices or where precision inhibitory titration is required. Integration into future food safety architectures is moving toward rational combinations, probiotic consortia + bacteriocins + mild physicochemical hurdles guided by multi‐omics and predictive ecology to prevent resistance, minimize sensory drift, and sustain shelf‐life extension. Figure 3 provides a schematic representation of probiotics and their derived antimicrobial compounds, illustrating their diverse applications in food safety and preservation, particularly in dairy products such as yogurt, cheese, and milk, where they contribute to improved product quality, safety, and extended shelf life. The potential application of probiotics and their metabolites in various food models is summarized in Table 1.

**TABLE 1:** Summarizes the bio‐preservation potential of probiotic and antimicrobial metabolites in various food models.

Food model/product	Probiotic(s) or Metabolite(s)	Dose/concentration	Key findings	References
Raw meat (beef and chicken samples)	*L. plantarum* , *P. acidilactici* , and *L. sakei* strains incorporated into marination liquids	Marination liquid contains approximately 10^8^ CFU/mL	Probiotic‐enriched marinades synergistically reduced *Listeria monocytogenes* , *E. coli* O157:H7, and *S. aureus* during storage without affecting meat quality, significantly extending shelf life.	Gargi and Sengun 2021
Fermented milk	*S. thermophilus* co‐cultured with *B. animalis* ssp. *lactis*, and *L. plantarum* , or both	Standard inoculation as per dairy starter culture protocols	Co‐culturing enhanced amino acids, organic acids, and bioactives, improving flavor, antioxidant potential, and metabolic diversity in fermented milk.	Li et al. 2021
Cooked minced beef	Freeze‐dried paraprobiotic of *P. acidilactici*	2%–4% (w/w) paraprobiotic added to meat model	*P. acidilactici* paraprobiotic markedly inhibited *L. monocytogenes* , *S. aureus* , and *E. coli* , preserving meat quality by reducing lipid oxidation and microbial spoilage during refrigerated storage.	İncili et al. 2023
Raw buffalo milk	LAB including *L. plantarum* , *L. fermentum* , *L. lactis* , and *E. faecium*	CFS of LAB (10%–100%) against foodborne pathogens	LAB isolates showed strong antimicrobial activity via bacteriocins, organic acids, and hydrogen peroxide, indicating potential as natural dairy biopreservatives.	Kalhoro et al. 2023
Milk	*L.s rhamnosus* RSQ01 (bacteriocin RSQ01)	Bacteriocin RSQ01 at 1× minimum inhibitory concentration (MIC)	Bacteriocin RSQ01 inhibited *Listeria monocytogenes* and *Staphylococcus aureus* , extending milk shelf life by suppressing amino acid and lipid degradation pathways.	Zhang et al. 2023
Ethiopian traditional fermented dairy products (Ergo, Ititu)	Bacteriocin‐producing *L. plantarum* , *L. lactis* , and *E. faecium* isolates	CFS containing bacteriocins (up to 1:4) against pathogens	Bacteriocinogenic LAB showed synergistic broad‐spectrum inhibition against major pathogens, highlighting strong potential for dairy bio‐preservation applications.	Amenu and Bacha 2024
Plant‐based meat model	*Lactobacillus brevis* C23 co‐culture producing γ‐aminobutyric acid (GABA) and bacteriocin‐like inhibitory substances (BLIS)	Crude postbiotic extract (10% v/v) incorporated into plant‐based medium	Co‐culturing elevated GABA and BLIS production, enhanced antimicrobial activity against *Listeria monocytogenes* and *Staphylococcus aureus* , prolonged shelf life, and preserved sensory quality.	Chuah et al. 2024
Red meat (surface decontamination model)	CSF of *L. rhamnosus* and *L. reuteri*	25%, 50%, and 75% (v/v) concentrations of postbiotic filtrates	Postbiotics effectively inhibited major pathogens and oxidative deterioration, enhancing meat quality, stability, and shelf life through antimicrobial and antioxidant activities.	Jalali et al. 2024
Fermented milk product	*Lactobacillus helveticus* CFS	CFS added at 10% (v/v) to fermented milk matrix	*L. helveticus* CFS enhanced fermented milk's antioxidant, antimicrobial, and peptide bioactivities, increased viable LAB counts, inhibited pathogens, and improved sensory and biological qualities.	Kishilova et al. 2024
Vacuum‐packed sliced emulsion‐type sausage	Cell‐free supernatant (CFS) of *Lactobacillus plantarum* , *Lactobacillus sakei* , and *Pediococcus pentosaceus*	CFS added at 10% (v/v) to inoculated meat model	CFS inhibited *B. thermosphacta* , *P. fluorescens* , and Enterobacteriaceae via bacteriocins, organic acids, and H_2_O_2_, enhancing sausage shelf life and safety without affecting sensory quality.	Tajbakhsh et al. 2024
Meat processing environment (surface and broth models)	Co‐culture of *L. sakei* CRL1384 and *E. faecium* CRL1831 (bacteriocinogenic LAB strains)	~10^7^ CFU/mL of each strain	Both strains inhibited *E. coli* O157:H7 planktonic and biofilm cells via organic acids, bacteriocins, and H_2_O_2_, disrupting metabolic and stress‐response proteins.	Cisneros et al. 2025
Frankfurters	*P. acidilactici* CFS; metabolites	2%–4% (v/w) in meat formulation	CFS rich in organic acids, peptides, and phenolics enhanced frankfurter oxidative stability, inhibited spoilage pathogens, and maintained sensory quality during storage.	İncili et al. 2025
Fresh pork (wrapped with probiotic fermented edible film)	*L. rhamnosus* incorporated into gum arabic/whey protein isolate/isomalt/glycerol‐based film	Viable cell count of ≈8.2 log CFU/g in the film matrix	The probiotic film enhanced mechanical and barrier properties, inhibited spoilage microbes, reduced lipid oxidation, and prolonged pork shelf life while preserving sensory quality.	Lu et al. 2025
Fresh meat model and Meat‐based model system	*L. plantarum* UTNGt21A and *L. plantarum* UTNGt2 and their metabolites (bacteriocins, organic acids, and biosurfactants)	CFS or postbiotic extract applied at 5%–20% (v/v) and	Postbiotic extracts showed broad‐spectrum antimicrobial activity, synergistic bactericidal enhancement, and stability under refrigeration, significantly reducing pathogens and improving microbiological safety without affecting sensory quality.	Molina et al. 2025

## 9 Beneficial Uses in Dairy Products: Yogurt, Cheese and Milk

The integration of probiotics and their derived antimicrobial compounds into dairy products represents the most advanced and commercially successful application in food preservation. Dairy products provide an ideal vehicle for probiotic delivery, offering a protective environment that enhances bacterial viability and functionality while simultaneously leveraging their antimicrobial activity to improve product safety and shelf‐life. Yogurt serves as a classic model for the symbiotic relationship between fermentation and bio‐preservation. Starter cultures ( *Streptococcus thermophilus* and *Lactobacillus delbrueckii* subsp. *bulgaricus*) acidify the milk, but the adjunct addition of probiotic strains, primarily from the genera *Lactobacillus* (e.g., *L. acidophilus* , *L. casei* ) and *Bifidobacterium* (e.g., *B. animalis* subsp. *lactis*), introduces a second layer of protection (García‐Burgos et al. 2020).

The probiotics used in fermented foods actively produce a diverse group of antimicrobial compounds, including organic acids (lactic and acetic acid), bacteriocins (nisin from *Lactococcus lactis* , lactacin B from *L. acidophilus* ), and bioactive metabolites generated during proteolysis (Moghanjougi et al. 2020). These compounds synergistically inhibit spoilage microorganisms and pathogens such as *Listeria monocytogenes* and *Escherichia coli* , thereby extending shelf life and enhancing food safety (García‐Burgos et al. 2020; Terpou et al. 2019). Empirical studies have qualified these effects; for example, the incorporation of *Lactobacillus casei* and *Bifidobacterium lactis* V9 (10^7^ CFU/mL each) in yogurt at 37°C, significantly delayed microbial spoilage, effectively extending the refrigerated shelf life from 14 to 21 days, while maintaining sensory and nutritional quality (Wang, Sun, et al. 2021; Wang, Zhao, et al. 2021). Similarly, supplementation of *L. plantarum* CCFM8661 and *B. animalis* BB‐12 (10^8^ CFU/mL each) in highland barley‐based yogurt significantly enhanced functional quality and shelf life up to 5–7 days primarily, due to the enhanced production of bioactive metabolites and phenolic compounds, with antimicrobial activity (Li et al. 2023). Moreover, the incorporation of *L. acidophilus* and *B. bifidum* (10^8^ CFU/mL each) into cow and goat milk yogurts enriched short‐chain fatty acids, amino acids, and organic acids, while both single and combined strains of *L. casei* Zhang and *B. animalis* ssp. lactis Probio‐M8 modulated volatile and nonvolatile metabolites (Wang, Sun, et al. 2021; Wang, Zhao, et al. 2021; Sharma and Ramanathan 2023). These studies indicate that probiotic‐driven metabolic reprogramming not only improves functional and nutritional quality but also provides a measurable, strain‐ and matrix‐dependent extension of shelf life in dairy and cereal‐based fermented foods, depending on strain composition, inoculum size, and storage conditions.

The application in cheese is multifaceted, involving the direct incorporation of probiotic cultures into the cheese matrix or the use of their purified bacteriocins as surface treatments. Probiotics, such as *Lactobacillus* spp. and *Bifidobacterium* spp., can be added during the curd formation stage. These probiotics have been shown to exert significant inhibitory effects against pathogenic microorganisms in cheese. Specifically, strains such as *Lactobacillus plantarum* and *Lactobacillus casei* can survive the cheese‐making process and persist during ripening, continuously producing antimicrobial metabolites like organic acid and bacteriocins that suppress contaminating pathogens and reduce surface spoilage (Ali et al. 2022). Silva, Balthazar, et al. (2018) demonstrated that incorporation of these probiotic strains in semi‐hard cheese resulted in notable reduction in *Staphylococcus aureus* counts, with growth suppression ranging from 1.5 to 3 log CFU/g over the ripening period, highlighting their practical antimicrobial potential in dairy matrices. Complementarily, Silva, Silva, and Ribeiro (2018) reported that applying bacteriocin‐enriched preparations, such as nisin‐based solutions, effectively inhibited the growth of *Listeria monocytogenes* on cheese surfaces, achieving reductions of up to 4 log CFU/g, thereby providing targeted protection in soft‐ripened and brined cheeses.

In fluid milk, the primary challenge is preserving freshness without the extensive fermentation used in yogurt. Probiotics are not typically used for preservation in fresh milk due to their acid‐producing nature, which would sour the product. Instead, the focus is on utilizing their purified antimicrobial proteins, particularly bacteriocins, as natural preservatives. For example, nisin is legally approved for use in pasteurized milk due to its strong activity against Gram‐positive post‐processing contaminants such as *Bacillus* and *Clostridium* spores that survive in thermal treatment (El‐Sayed et al. 2022). Recent research explores the synergistic effect of combining bacteriocins with non‐thermal technologies like high‐pressure processing (HPP) also control Gram‐negative bacteria, offering a potential hurdle technology strategy to extend the shelf‐life of pasteurized milk without compromising its sensory properties (Sikin et al. 2017). In conclusion, the strategic use of probiotics and their antimicrobial metabolites in dairy products effectively creates a resistance against spoilage and pathogens. This not only enhances food safety particularly against post‐processing contaminants but also aligns with the growing consumer demand for chemical free, natural preservation methods.

## 10 Beneficial Uses in Meat and Poultry Products

Ensuring microbiological safety, extended shelf life, and preserving the quality of fresh and processed meat products, including poultry, remains a key priority for the food industry to provide consumers with safe, hygienic, and high‐quality meat (Arain, Hassan, et al. 2025). The increasing shift toward minimally processed and clean‐label foods has driven interest in strategies as natural alternatives to synthetic antimicrobials (Arain et al. 2018, 2022). Among these probiotic cultures and their AMPs, particularly bacteriocins, have emerged as promising tools due to their ability to suppress pathogenic and spoilage microorganisms while maintaining product quality (Lorenzo et al. 2018). It is important to clarify, however, that both probiotics and bacteriocins can be used individually or sequentially for meat bio‐preservation; current literature does not provide conclusive evidence of true synergistic antibacterial effects specifically between co‐applied probiotics and bacteriocins in meat systems. Most studies either apply live protective cultures that act via competitive exclusion and in situ metabolite production, or they examine the direct incorporation of purified bacteriocins as standalone hurdles. LAB represent the most extensively studied group for meat preservation. Strains such as *Lactobacillus sakei* , *Lactobacillus plantarum* , *Pediococcus acidilactici* , and *Lactococcus lactis* have demonstrated strong inhibitory effects against spoilage and pathogenic organisms. When applied as starter or protective cultures in fermented sausages like salami, LAB produce lactic acid that lowers pH, thereby suppressing *Listeria monocytogenes* and *Staphylococcus aureus* (Fernández et al. 2023). Pirnia et al. (2025) demonstrated that chitosan‐based coatings enriched with *Lactiplantibacillus plantarum* postbiotics significantly inhibited spoilage bacteria, including *Pseudomonas* spp. and *Enterobacteriaceae*, in sausages. This approach effectively prolonged chilled storage life while maintaining microbial safety and sensory quality, highlighting probiotic‐enriched coatings as a clean‐label strategy for meat preservation (Li et al. 2025).

The most prominent role of probiotics in preservation is through bacteriocin production. These peptides exhibit strong antimicrobial activity, particularly against Gram‐positive bacteria. Nisin, produced by *L. lactis* , and pediocin, from *Pediococcus* spp., are well‐characterized bacteriocins with commercial applications. Their primary mechanism involves pore formation in microbial cell membranes, which effectively inhibits *L. monocytogenes* a persistent threat in ready‐to‐eat meat products (Ramu et al. 2015). Direct application of bacteriocins, such as embedding nisin into active packaging films, has proven effective in suppressing *L. monocytogenes* on vacuum‐packed turkey breast and cooked ham (Gutiérrez 2010). Combining probiotics or bacteriocins with other preservation technologies enhances their antimicrobial efficacy. This approach exploits synergies between biopreservatives and sub‐lethal interventions like high‐pressure processing (HPP), modified atmosphere packaging (MAP), or natural extracts. For example, HPP disrupts bacterial membranes, rendering pathogens more susceptible to bacteriocins, thereby ensuring enhanced microbial safety while maintaining sensory quality (Singh and Shalini 2016).

Recent studies have expanded the scope of probiotics beyond preservation to poultry production systems, where they serve as alternatives to antibiotic growth promoters. Supplementation with probiotics and antimicrobial peptides improved growth performance, gut morphology, immune function, and carcass quality in broilers challenged with *Eimeria* and *Clostridium perfringens* (Muneeb et al. 2025; Arain, Khaskheli, et al. 2024). Similarly, dietary supplementation with *Bacillus licheniformis* enhanced broiler growth, hematological parameters, and meat quality, supported by improved protein and lipid metabolism (Khan et al. 2025). Although numerous studies have demonstrated the individual application of either probiotics or bacteriocins in meat preservation, direct experimental evidence confirming their synergistic interaction in meat matrices remains limited. For example, probiotics have been shown to improve oxidative stability and preserve sensory attributes of chicken meat during storage (Gharaghani et al. 2013). In parallel several bacteriocins exhibit strong bio‐preservative effects against major meat‐borne pathogens. Amylolysin, from *Bacillus amyloliquefaciens GA1* effectively inhibited *L. monocytogenes* in poultry meat (Halimi et al. 2010), while *L. plantarum* BFE 5092 suppressed both spoilage and pathogenic bacteria in raw turkey meat (Cho et al. 2010). In fresh beef systems, probiotic incorporation reduces lipid and protein oxidation and improves color and texture stability (Trabelsi et al. 2019). Similarly, probiotic‐based yogurt marinades enhance microbial safety, pH balance, texture and sensory quality in chicken filets (Masoumi et al. 2022).

LAB and their metabolites including organic acids and bacteriocins provide substantial bio‐preservation benefits, offering clean‐label alternatives to synthetic additives (Kaveh et al. 2023). For example, *Lactiplantibacillus plantarum* s61 inhibited *Rhodotorula glutinis* and *L. monocytogenes* in poultry meat, confirming its potential preservative efficacy (Abouloifa et al. 2022). However, while both probiotics and bacteriocins independently enhance meat safety and quality, current literature lacks mechanistic or empirical studies validating a synergistic preservative effect when both are co‐applied within the same system. Future research should focus on controlled co‐application models and mechanistic elucidation to determine whether combined use offers additive or synergistic advantages beyond their individual performance.

## 11 Beneficial Uses in Seafood

The application of probiotics and their bioactive metabolites, particularly bacteriocins and other AMPs, has emerged as a sustainable strategy to extend shelf‐life and enhance the safety of seafood products. LAB, including *Lactobacillus*, *Pediococcus*, and *Carnobacterium* spp., are widely used as protective cultures in seafood biopreservation. When inoculated onto fish or shellfish, they competitively inhibit spoilage and pathogenic microorganisms such as *Listeria monocytogenes* and *Pseudomonas* spp. through nutrient competition, production of organic acids, hydrogen peroxide, and AMPs (Ghanbari et al. 2013). Biopreservation using probiotic cultures has been shown to reduce total viable counts and specifically suppress *Shewanella putrefaciens* , the primary cause of off‐odors in spoiled fish, thereby delaying spoilage (Pilet and Leroi 2011). The most potent effects are often linked to bacteriocins such as nisin, pediocin, and carnobacteriocin, which display broad or narrow antimicrobial activity against Gram‐positive pathogens, notably *L. monocytogenes* and *Clostridium botulinum* (Pérez‐Sánchez et al. 2011). Their incorporation into edible coatings and packaging materials has further enhanced their efficacy. For instance, chitosan‐based coatings enriched with nisin effectively controlled microbial growth on fish filets, with synergistic effects from bacteriocin with other bioactive, ensuring prolonged antimicrobial action at contamination‐prone surfaces (Derakhshan‐Sefidi et al. 2024). Purified bacteriocin extracts and other AMPs can be applied directly, eliminating concerns regarding microbial viability and offering enhanced stability in seafood systems (Iseppi et al. 2021). In this context, *Paenibacillus provencensis* metabolites (organic acids, bacteriocins, and small bioactive compounds), significantly inhibited the growth of spoilage pathogenic microorganisms and suppressed lipid oxidation in refrigerated sea bass ( *Perca fluviatilis* ), thereby preserving freshness and extending shelf‐life (Liu et al. 2025). Similarly, *Bacillus subtilis* inhibited *Pseudomonas* spp. adhesion and biofilm formation, significantly reducing spoilage in fresh fish (Zhang et al. 2021).

Recent metabolomic evidence indicates that specific LAB strains ( *Leuconostoc citreum* M8 (M8)) effectively prolonged discoloration in tuna filets by increasing the levels of key antioxidant metabolites, including glutathione, carnosine, and ascorbate, which help neutralize reactive oxygen species and stabilize myoglobin pigments, thereby maintaining the meat color (Jo et al. 2025). Likewise, *Lactobacillus rhamnosus* exhibited strong antagonistic activity against both native and specific spoilage bacteria in Asian seabass, significantly reducing microbial load and spoilage indices (Kannappan et al. 2017). Bacteriocinogenic LAB, particularly strains belonging to *Lactobacillus plantarum, L. acidophilus, L. delbrueckii, L*. *brevis* , *L. rhamnosus, Enterococcus faecium*, and *Pediococcus acidilactici* , have been consistently identified as potent producers of AMPs or bactericins. These bacteriocins exhibit broad‐spectrum inhibitory activity against foodborne and spoilage microorganisms (Hwanhlem and H‐Kittikun 2015). Advances in food packaging have facilitated the integration of probiotic‐derived metabolites into bioactive films. Basil mucilage and cellulose nanofiber‐based edible films enriched with postbiotics effectively delayed microbial spoilage, lipid oxidation, and sensory deterioration in fish filets (Mokhtaran et al. 2025). Additionally, *Leuconostoc mesenteroides* J.27‐derived postbiotics combined with food‐grade essential oils exhibited strong antibiofilm activity against *Vibrio parahaemolyticus* , *Pseudomonas aeruginosa* , and *Escherichia coli* , with enhanced effects in synergistic applications (Toushik et al. 2022). Beyond bacteriocins, phenyl lactic acid (PLA) has demonstrated potent antibacterial activity against *Aeromonas hydrophila* by disrupting cell membrane integrity, elevating reactive oxygen species, and impairing bacterial metabolism. Its supplementation improved fish disease resistance and extended fish product preservation (Xia et al. 2025). Collectively, probiotics and their secreted antimicrobial compounds particularly bactericins and metabolites represent eco‐friendly, and effective bio‐preservation strategic solutions for seafood. These bioactive agents can be applied through probiotic inoculation or by incorporating their purified antimicrobial components into preservation systems, thereby improving microbial safety, extending shelf‐life, and maintaining product quality.

## 12 Technological Advances for the Application of Probiotics and Their AMPs in Food Preservation

The application of probiotics and their AMPs, particularly bacteriocins, in the food industry faces major challenges due to their instability under processing and gastrointestinal conditions. To address these limitations, innovative technologies have been developed to improve their stability, viability, and targeted release, thereby enhancing their effectiveness in food preservation and functional food development.

Microencapsulation has emerged as a leading strategy to protect probiotics and bacteriocins against harsh processing environments and gastric acidity. Techniques such as spray‐drying, extrusion, and emulsion templating enclose live probiotic cells within protective matrices composed of alginate, chitosan, or whey proteins. This significantly improves probiotic survival during storage and transit through the gastrointestinal tract, while enabling controlled release at the target site. Similarly, bacteriocins such as nisin and pediocin can be encapsulated to prevent interactions with food components and extend their antimicrobial activity against spoilage and pathogenic microorganisms (Bagheri Darvish et al. 2021; Soccol et al. 2017).

Another promising approach is the immobilization of probiotics and AMPs in edible films and coatings, which creates a protective layer on food surfaces. Biopolymer‐based films derived from starch, cellulose, or alginate not only regulate moisture loss and gas exchange but also act as carriers for antimicrobials. These coatings continuously release bacteriocins or probiotic metabolites, effectively inhibiting surface contamination in products such as cheese, fresh‐cut fruits, and meats. By integrating antimicrobial metabolites directly into packaging, this approach reduces reliance on chemical additives traditionally used for food preservation, such as synthetic antioxidants, preservatives, and antimicrobials, while simultaneously enhancing product safety (Hassan et al. 2018). This strategy leverages naturally produced bioactive metabolites such as organic acids, bacteriocins and antimicrobial proteins from probiotics or other microbial sources, which inhibit spoilage and pathogenic microorganisms without compromising sensory and nutritional quality. Moreover, packaging films embedded with bacteriocins or probiotic‐derived biosurfactants, for example, can selectively inhibit *Listeria monocytogenes* in ready‐to‐eat foods without affecting sensory quality (Kourmentza et al. 2021). Compared to conventional chemical preservatives, antimicrobial metabolites offer targeted microbial control, lower toxicity risks, and improved consumer acceptability, making them a sustainable and effective alternative for extending shelf life in food products.

The most advanced frontier in this field lies in nanoformulations and delivery systems. Nanoencapsulation using liposomes, solid lipid nanoparticles (SLNs), and nanoemulsions provides superior protection and precision release compared to traditional microencapsulation. Their large surface‐area‐to‐volume ratio enhances the dispersion and antimicrobial efficacy of bacteriocins at lower concentrations. Techniques like electrospinning enable the development of nanofibrous mats containing probiotics for intelligent packaging, while nanoemulsions of bacteriocins serve as highly efficient surface sanitizers. These nanoscale delivery systems represent a transformative advancement, allowing for precise and responsive antimicrobial activity tailored to food preservation needs (Lavanya et al. 2024).

## 13 Challenges and Limitations

The translation of in vitro efficacy of probiotics and AMPs, particularly bacteriocins, into food preservation systems faces multiple challenges across technological, regulatory, and consumer dimensions. Ensuring probiotic viability and AMP stability during processing and storage is a major hurdle. Thermal treatments (e.g., pasteurization, baking), high‐pressure processing, and exposure to pH extremes or oxygen can denature proteinaceous AMPs and reduce viable cell counts (Linares‐Morales et al. 2018). Additionally, interactions with food components such as fats, proteins, and enzymes can inactivate AMPs, while storage conditions involving temperature fluctuations or acid accumulation impair probiotic survival (Suez et al. 2019). Encapsulation and strain engineering offer solutions but increase technological complexity and cost.

Effective antimicrobial activity is strongly influenced by the food matrix. Parameters such as pH, water activity, fat content, and the resident microbiota affect the minimum effective dose. Suboptimal dosing may reduce efficacy, whereas higher concentrations risk altering sensory properties like flavor or texture (Garcia‐Gutierrez et al. 2020). Moreover, not all probiotic strains produce AMPs effective against relevant spoilage or pathogenic organisms, making strain‐specific screening and validation essential, though resource‐intensive. On the other hand, regulatory approval of probiotics or AMPs as food preservatives is complex and region‐specific. Probiotic strains require evidence of safety, such as Qualified Presumption of Safety (QPS) status in the EU, while purified AMPs are often categorized as food additives requiring toxicological evaluations and acceptable daily intake (ADI) determination (Huang et al. 2021). Concerns also exist regarding horizontal transfer of AMP‐associated resistance genes, necessitating thorough genetic safety assessments. Finally, the market success of probiotics and AMPs depends heavily on consumer perception. Terminology such as “bacteriocins” may raise skepticism, whereas alternative descriptors like “natural bioprotective cultures” are more acceptable (Todorov et al. 2022). Furthermore, clean‐label preferences and dietary considerations, particularly among vegan and vegetarian consumers, underscore the need for transparent and culturally sensitive labeling practices.

## 14 Future Perspectives

Probiotics and their antimicrobial metabolites, particularly bacteriocins, are emerging as promising natural tools for sustainable food preservation. Advances in genetic engineering now enable the construction of probiotic strains that overproduce and secrete next‐generation AMPs with broadened target ranges, including resistant foodborne pathogens. Synbiotic formats combining probiotics, prebiotic substrates, and purified AMPs are also anticipated to create self‐sustaining inhibitory microenvironments within food matrices, extending shelf life with minimal formulation changes. Beyond live cultures, postbiotics inanimate microbial cells or metabolites with proven bioactivity are attracting interest as safer, more stable, and friendly alternatives for incorporation into commercial foods. The key technical challenge has been the instability and low bioavailability of AMPs and live cultures in complex food systems (Suez et al. 2019). Emerging nano‐delivery platforms (e.g., solid lipid nanoparticles, polymeric encapsulates, nanofibrous matrices) provide protection, targeted release, and improved persistence, marking a major leap toward functional deployment (Abaidullah et al. 2025). In parallel, research is shifting from single‐strain applications to rationally designed consortia that co‐produce multiple AMPs with complementary modes of action, reducing the risk of resistance evolution. Collectively, these developments position probiotic‐derived AMPs, engineered consortia, synbiotics, and postbiotic formulations as core components of next‐generation, food preservation frameworks.

## 15 Conclusion

This review highlights the complementary role of probiotics and their antimicrobial metabolites, in food preservation, emphasizing not only competitive exclusion and immune modulation but also the potential contributions of other bioactive metabolites such as organic acids and hydrogen peroxide. These metabolites enhance antimicrobial efficacy by lowering pH, generating oxidative stress, and disrupting pathogen metabolism, thereby reinforcing the inhibitory action of secreted proteins. The integration of these bioactive compounds creates a multi‐layered defense system that improves food safety, extends shelf life, and maintains nutritional and sensory qualities. Beyond microbial control, this dual strategy contributes to conferring added health benefits to consumers, aligning with the growing demand for “clean‐label” and natural food products. Despite these advantages, translating this synergy into industrial applications requires vigilant consideration of strain‐specific metabolite production, stability under processing conditions and potential effects on flavor and texture. Advances in strain engineering, encapsulation, and controlled release technologies, combined with rigorous safety evaluations and regulatory compliance are critical for optimizing the efficacy and consumer acceptance of such natural preservation strategies.

## Author Contributions

**Lingling Wang:** conceptualization, data curation, validation. **Shuanshan Ren:** conceptualization, validation, writing – original draft. **Atique Ahmed Behan:** formal analysis, visualization, writing – review and editing. **Muhammad Asif Arain:** writing – review and editing, visualization, methodology, data curation. **Nissar Ahmed Ujjan:** software, validation, writing – review and editing. **Dequan Zeng:** methodology, visualization, writing – review and editing. **Yufeng Li:** data curation, investigation, writing – review and editing. **Xingming Ma:** writing – original draft, validation, conceptualization. All authors read and approve the final draft for publication.

## Funding

The authors acknowledged their financial support was received for the research and/or publication of this article. This study was supported by the Sichuan Province Science and Technology Department project (grant number: 2024NSFSC1750).

## Ethics Statement

The authors have nothing to report.

## Conflicts of Interest

The authors declare no conflicts of interest.

## Data Availability

The data that support the findings of this study are available from the corresponding author upon reasonable request.
